# A Review of the Epidemiology of Breast Cancer in Asia: Focus on Risk Factors 

**DOI:** 10.31557/APJCP.2020.21.4.867

**Published:** 2020-04

**Authors:** Hyun Jo Youn, Wonshik Han

**Affiliations:** 1 *Department of Surgery, Research Institute of Clinical Medicine, Chonbuk National University and Biomedical Research Institute, Chonbuk National University Hospital, *; 2 *Department of Surgery and Cancer Research Institute, Seoul National University College of Medicine, Seoul National University Cancer Hospital, Republic of Korea. *

**Keywords:** Breast neoplasms, epidemiology, Asia, risk factors

## Abstract

**Background and Aim:**

Breast cancer is the most prevalent cancer in women. To date, regional differences in breast cancer risk factors have not been identified. The aim of our review was to gain a better understanding of the role of risk factors in women with breast cancer in Asia.

**Methods:**

We conducted a PubMed search on 15 March 2016, for journal articles published in English between 2011 and 2016, which reported data for human subjects in Asia with a diagnosis of breast cancer. Search terms included breast neoplasm, epidemiology, Asia, prevalence, incidence, risk and cost of illness. Studies of any design were included, except for review articles and meta-analyses, which were excluded to avoid duplication of data. No exclusions were made based on breast cancer treatment. We reported the results using the Preferred Reporting Items for Systematic Reviews and Meta-analyses (PRISMA) guidelines.

**Results:**

A total of 776 abstracts were retrieved. After screening against the eligibility criteria, 562 abstracts were excluded. The remaining 214 abstracts, which were published between 2013 and 2015, were included in this review. Results were summarized and reported under three categories: incidence, prevalence or outcomes for breast cancer in Asia; modifiable risk factors; and non-modifiable risk factors. We found that the increased risk of breast cancer among participants from Asia was associated with older age, family history of breast cancer, early menarche, late menopause, high body mass index, being obese or overweight, exposure to tobacco smoke, and high dietary intake of fats or fatty foods. In contrast, intake of dietary fruits, vegetables, and plant- and soy-based products was associated with a decreased breast cancer risk. While based on limited data, when compared to women from the United States, women from Asia had a decreased risk of breast cancer.

**Conclusions:**

This review of 214 abstracts of studies in Asia, published between 2013 and 2015, confirmed the relevance of known non-modifiable and modifiable risk factors for women with breast cancer.

## Introduction

The most recent data from the World Health Organization (WHO) shows that breast cancer (BC) is the most prevalent cancer in women, with an incidence of 1.7 million cases in 2012 (25 % of all female cancers). It is the fifth leading cause of death from cancer overall (International Agency for Research on Cancer, 2016). The incidence is similar in regions identified by the WHO as more- and less-developed, at 13% (788,000) and 11% (883,000) respectively (International Agency for Research on Cancer, 2016). WHO figures from 2012 estimate the international 5-year prevalence for BC at 6,232,000; by region (as defined by WHO) the rate in South-East Asia is 735,000 compared to 1,936,000 in Europe and 1,618,000 in the Americas (International Agency for Research on Cancer, 2016).

Identified risk factors for BC may be non-modifiable such as sex, age, genetic characteristics including family or personal history of BC, ethnicity, and early menarche or menopause. Modifiable risk factors, usually associated with lifestyle factors, can include alcohol consumption, excess weight or obesity, physical inactivity, parity, and use of some medications, such as oral contraceptives (American Cancer Society, 2016; World Health Organization, 2016).

Despite these associations, women with risk factors for BC may never go on to develop BC and many women with BC have no known risk factors. Therefore, it is difficult to definitively determine the contribution of individual risk factors to the development of BC (American Cancer Society, 2016). 

To date, regional differences in BC risk factors have not been identified. The aim of our review was to gain a better understanding of the role of risk factors in women with BC in Asia.

## Materials and Methods

We performed a systematic review of the current body of evidence available through PubMed. The search was conducted on 15 March 2016. Medical subject headings (MeSH) search terms used included: breast neoplasm, epidemiology, Asia, prevalence, incidence, risk and cost of illness. The search was limited to human studies, published in English between 2011 and 2016. Studies were included in our review if the participants had BC of any age, histopathology, grade or stage, and received treatment of any type; studies needed to report information on epidemiology, prevalence, incidence, risk factors, and cost of illness. Our review included all study designs, except for review articles and meta-analyses, which were excluded to avoid duplication of data. Studies were only included if they reported data from countries within South East Asia, Far East Asia, or Western Asia excluding the Middle East. Articles were excluded if their primary objective was to examine the correlation of BC to other types of cancer or to examine surgical treatments for BC. 

## Results

**Figure 1:**
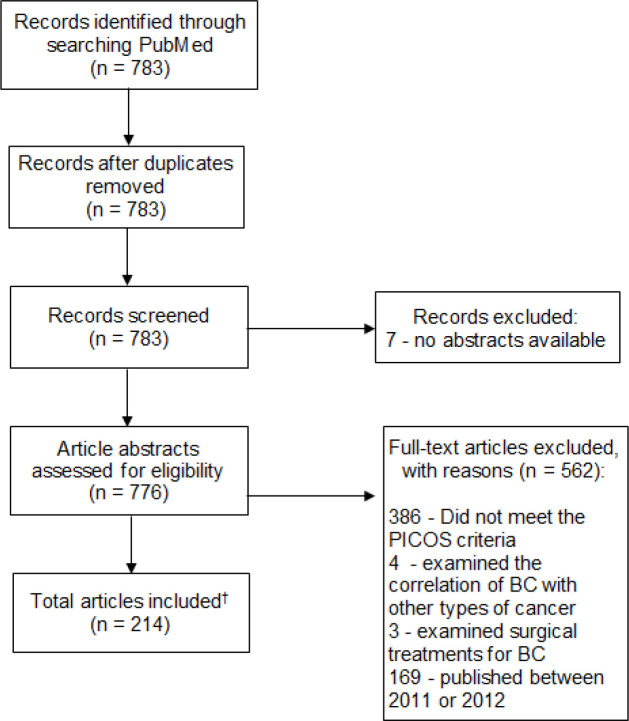
Flow Diagram of the Literature Selection Process. NA, not applicable; * No other sources were searched; †, Due to the volume of data available for analysis, results were analyzed based only on the content of study abstracts; BC, breast cancer

Our search identified 783 articles; seven articles were excluded due to no abstract being available. We did not find any articles published in 2016. To reduce the volume of data identified in our search, only articles published within the previous three years (2013–2015) were included. This resulted in 169 eligible articles being excluded based on publication date (2011 and 2012). After the exclusion of duplicates and articles that did not meet the inclusion criteria, a total of 214 remaining articles were included in this review. The flow diagram of the literature selection process is presented in Figure 1.

**Figure 2.**
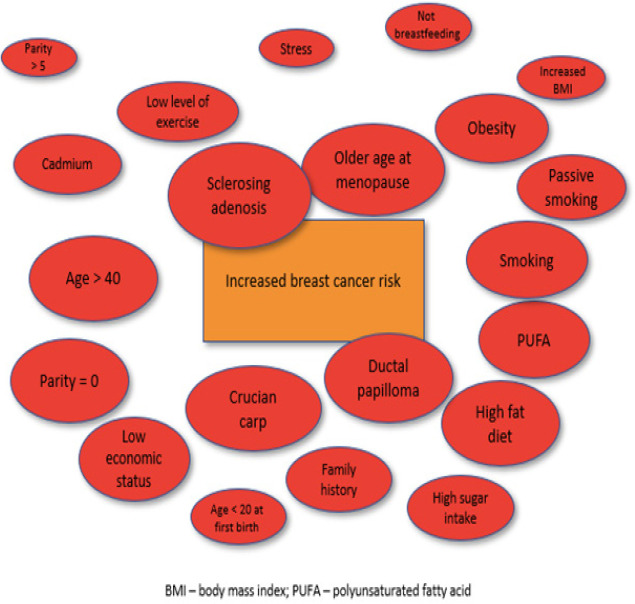
Risk Factors for Breast Cancer in Asia

**Figure 3:**
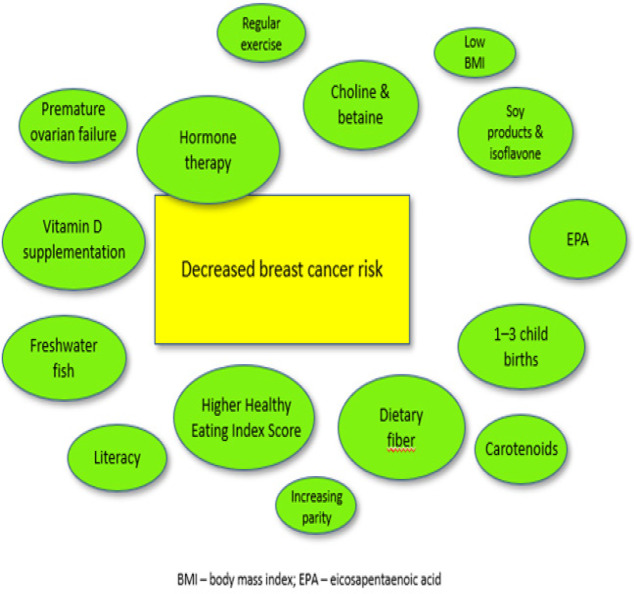
Protective Factors for Breast Cancer in Asia

Results have been summarized and reported under three categories: incidence, prevalence or outcomes for breast cancer in Asia; modifiable risk factors; and non-modifiable risk factors. A summary of studies showing risk and protective factors influencing changes in BC risk is provided in Supplementary Table 2; a visual representation of risk and protective factors is provided in Figure 2 and Figure 3. Due to the large number of studies eligible for review, descriptive data without an association with altered BC risk have not been provided in the tables. Key trends are summarized in the sections below.

*Incidence, prevalence or outcomes for breast cancer in Asia*

BC was reported as the leading type of cancer in females in Japan (Matsuda et al., 2014; Hori et al., 2015), and China (Zhou et al., 2015). In addition, the incidence of BC was shown to increase over time in China (Wu et al., 2014b; Zhou et al., 2015), India (Asthana et al., 2014) and Thailand (Virani et al., 2014), although it was unclear if this was due to improved identification (i.e. screening and detection) or a true reflection of increased incidence. While BC incidence is predicted to increase in Hong Kong by 2025, mortality is projected to decrease from 9.3 to 8.6 per 100,000 women, respectively (Wong et al., 2015). This may be related to Hong Kong’s socio-economic development, and the improvements in survival may be due to treatment advancement and improved health service delivery (Wong et al., 2015). This is in contrast to China, where BC mortality increased over a 5-year period (annual percentage change of 6.89%) between 2006 and 2010 (Wu et al., 2014b). In one study from Malaysia, almost all (n=1453, 99.2%) had an estimated 10-year risk of BC of less than 2% (Hassan et al., 2015). In Korea, the rate of 5-year recurrence-free survival (RFS) was 90.8%, 10-year RFS was 81.9%, and 5-year overall survival (OS) was 94.6% (Kim et al., 2014; Park et al., 2015b). However other studies from Korea have reported the 10-year OS rate at 86.9% (Park et al., 2015b) and another at 61.4% (Jung et al., 2013). In Hong Kong and Vietnam, 5-year OS rates were 90.5% and 74%, respectively (Lan et al., 2013; Kwong et al., 2014). The 5-year OS rate was similar in male BC patients from Hong Kong (n=132, 78.7%) (Kwong et al., 2014). Supplementary Figure 1 Flow diagram of the literature selection process

*Dietary Factors*

Dietary factors associated with increased BC risk included intake of total n-6 polyunsaturated fatty acids (PUFA) (Kiyabu et al., 2015), vitamin D deficiency or insufficiency (Shi et al., 2014a; Park et al., 2015a), high levels of serum cadmium (Nagata et al., 2013; Itoh et al., 2014; Ding et al., 2015), high intake of salt (Park et al., 2014b), sugar (Sulaiman et al., 2014), meat (Ko et al., 2013), and saturated fat and oils (Balasubramaniam et al., 2013; Wang et al., 2013a). In women with BC, the trace elements, cadmium, magnesium, copper, cobalt and lithium, were found in high levels. This suggests a possible association between serum levels of trace elements and breast cancer risk, however, this requires further investigation and if confirmed, the modulation of trace elements may help to reduce breast cancer risk (Ding et al., 2015).

High intake of dietary fiber was associated with decreased BC risk (Sulaiman et al., 2014) along with high levels of α-carotene, β-carotene, β-cryptoxanthin, lutein/ zeaxanthin (Wang et al., 2014a), and soy products or isoflavones (Ko et al., 2013; Li et al., 2013a; Wada et al., 2014) (Table 2). In addition, BC patients had significantly lower levels of manganese, aluminum, iron and titanium than women who did not have BC (Ding et al., 2015). High consumption of fruit and vegetables has also been linked to a reduction in breast cancer risk in Asia. (Sangrajrang et al., 2013)

In one study, the consumption of crucian carp was associated with increased BC risk, but total freshwater fish intake and consumption of black carp and silver carp were associated with decreased risk (Gao et al., 2014).

*Location of residence*

Location of residence were identified as a modifiable factor associated with increased BC risk and was identified in three papers (Shi et al., 2014b; Wu et al., 2014b; Fei et al., 2015). Results of the studies identified that living in an urban region of China (versus a rural region) led to an increased BC risk. One paper noted a higher incidence of BC was correlated to higher population density, percentage of non-agricultural population, and second industry output; in contrast, incidence was negatively correlated with the percentage of population employed in primary industry (Fei et al., 2015). Other modifiable risk factors including active or passive smoking (Mizoo et al., 2013; Pimhanam et al., 2014; Tong et al., 2014; Wada et al., 2015), high body mass index (BMI) (Mizoo et al., 2013; Sangrajrang et al., 2013; Suzuki et al., 2013; Wang et al., 2013b; Anothaisintawee et al., 2014; Wada et al., 2014; Fu et al., 2015), use of oral contraceptives (Bhadoria et al., 2013; Anothaisintawee et al., 2014; Poosari et al., 2014), high perceived level of stress, and low level of physical activity (Wang et al., 2013a) were discussed in the papers.

*BMI and physical activity*

A Thai study evaluated the relationship between BC risk and obesity and physical activity. The study found that being underweight at ages 10 and 20 years showed a reduced risk of BC in all women (OR=0.70, 95%CI 0.56-0.88 and OR=0.74, 95%CI 0.59-0.93, respectively) and undertaking regular physical activity also showed a reduced risk (OR=0.78, 95% CI 0.68-0.98). When reviewed by type of physical activity undertaken, those who had the highest levels of walking had the greatest reduction in risk (OR= 0.58, 95% CI 0.38-0.88) (Sangrajrang et al., 2013). 

*Reproductive history and breastfeeding*

Several of the identified studies have investigated the effect of parity, abortion, age at pregnancy and history of breastfeeding on BC risk. In a Korean study, parity of five or more increased the risk of luminal A and B breast cancer (HR 1.95, 95% CI, 0.96-3.97, p=0.0055; and HR 1.12; 95% CI, 0.42-3.02, p=0.0073 respectively) and an early age of first birth (less than 20 years) was associated with increased risk of luminal A BC recurrence (HR 1.61, 95% CI, 0.62-4.26; p=0.039). However, between one and three childbirths was associated with decreased risk of luminal A and B (HR, 0.56; 95% CI, 0.34-0.91, p= 0.0055; and HR, 0.32; 95% CI, 0.17-0.61, p=0.0073 respectively) (Lee and Oh, 2014). In India, women with BC, had a higher age at last child birth, lower mean duration of breastfeeding, and a higher number of abortions compared to women who did not have BC (Bhadoria et al., 2013). In China, Wu et al., (2014a) reported reduced BC risk in parous women with 3 or more induced abortions or 3 or more induced abortions after first live birth, compared with parous women without induced abortion. Also in China, age over 25 years at first full-term pregnancy was associated with increased risk of BC, but a history of full-term pregnancy was associated with a decreased risk of BC (Pei et al., 2014). Huang et al., (2014) reported that among participants from China, later age at either first live birth, or at first pregnancy and last pregnancy were associated with increased BC risk (p-trend=0.002, 0.015, 0.008, respectively). Among participants from Japan, BC was increased in women who performed mixed feeding (HR 1.12, 95% CI, 0.92-1.37, p-trend=0.014) or feeding only with formula (HR 1.80, 95% CI, 1.14-2.86, p-trend=0.014) compared with women who only breastfed (Sugawara et al., 2013).

*Breast cancer risk by geographic location*

Several studies demonstrated differences in the prevalence and mortality associated with BC between nations in the Asian region, and significantly, differences within individual nations. Ginsburg et al., (2017) argue that the majority of women who die from BC live in low-income countries; this is in contrast to high-income countries where most women who are diagnosed with BC will survive. Malvia et al., (2017) indicate that the overall age adjusted rates for BC in India is as high as 25.8 per 100,000 women but lower than that of the United Kingdom (95 per 100,000). Indian mortality rates were reported at 12.7 per 100,000 women, which is comparable to the BC mortality rates in the UK (17.1 per 100,000). Further, disparities in BC risk and mortality can be seen within individual countries in Asia and within different regions within the same country. When Indian BC mortality rates are reported by region, some regions reported rates as high as 41 per 100,000 in Delhi and as low as 12.4 per 100,000 in Barshi rural region (Malvia et al., 2017). 

*Non-modifiable risk factors for breast cancer in Asia Age*

In India, the mean age of BC diagnosis was 53 years (Tulsyan et al., 2014), and the median age was 45 years (Krishnatreya et al., 2014). This was similar to data from China, which indicated that the estimated risk of recurrent breast cancer increased with age (mean=46 years) (Chen et al., 2014b). Peaks in age-specific breast cancer incidence were at 50–59 years and > 85 years (Wu et al., 2014b). In a Thai study, incidence was projected to increase at a higher rate in women aged 50 years or over compared to those aged under 50 years (period to 2029) (Virani et al., 2014). Other studies have reported that benign breast disease was more likely to occur in women aged under 40 years, with BC more likely in women aged over 40 years (Kotepui and Chupeerach, 2013; Kotepui et al., 2014). 

*Family history*

In China (Zhou et al., 2013; Chen et al., 2014b; Lee et al., 2014; Pei et al., 2014), Pakistan (Shamsi et al., 2013), Hong Kong (Tse et al., 2015), India (Bhadoria et al., 2013), and Korea (Park et al., 2014a), a family history of BC was associated with an increased risk of BC. In Hong Kong, risk of BC (in women with estrogen receptor-positive tumors) was higher in women with an affected mother (OR = 3.97, 95%CI: 1.46–10.79) when compared to women with an affected sister (OR = 2.06, 95%CI: 1.07–3.97) (Tse et al., 2015). A study in Nepal showed that family history of BC was evident in a higher proportion of women who developed BC aged less than 40 years (3%) compared with those aged 40 years or over (0.3%) (Thapa et al., 2013).

*Ethnicity*

Race/ethnicity and age-specific incidence rates have been shown to increase continuously until 80 years in white women from the United States (US), but plateau or decrease after 50 years in Asian women (Sung et al., 2015). A greater absolute risk for all BC subtypes has been found in women from the US National Cancer Institute’s Surveillance, Epidemiology, and End Results 18 registries database (SEER 18) compared to those from Malaysia (Horne et al., 2015). A further study demonstrated that, following adjustment for population structure, the median age of BC diagnosis was lower in China (50–54 years) than in the United States and European Union (55–59 years) (Song et al., 2014b). The authors suggest that this is due to racial differences in genetics and lifestyle and that BC screening programs should be commenced at an earlier age for Chinese women (Song et al., 2014b).

*Reproductive health*

Menopausal status was associated with BC risk in studies conducted in Thailand (Anothaisintawee et al., 2014), India (Chattopadhyay et al., 2014), Pakistan (Sohail et al., 2013), and Korea (Park et al., 2013). Among participants from India, compared with controls, women with BC had a lower mean age at menarche (13.20±1.33 and 14.58±0.85 respectively, p=0.00) and a later age of menopause (49.38±5.21 and 47.89±3.96 respectively, p=0.002) (Bhadoria et al., 2013). Lee et al., (2014) reported that late menopause was significantly associated with increased BC risk. In Korea, age at menarche and menopausal status or age at menopause were risk factors for BC (Park et al., 2013). A second study in Korea confirmed the association with early age at menarche and BC risk, irrespective of tumor subtype (Chung et al., 2013). In Pakistan, older age at menopause was associated with increased BC risk (Shamsi et al., 2013). 

*Hormone receptor status*

In China, estrogen levels and estrogen receptor-mediated serum bioactivity were higher in BC cases than controls (Lim et al., 2014). A study conducted in Malaysia found that the majority of BC cases were hormone receptor-negative (Horne et al., 2015). Conversely in a Korean study, 69.5% of BCs were hormone receptor-positive (Song et al., 2014a) and 44.6% of BCs were estrogen receptor-positive in an Indian study (Singh et al., 2014). Among participants from Korea, when estrogen receptor-positive/progesterone receptor-positive tumors were compared to estrogen receptor-positive/progesterone receptor-negative tumors, women with the latter were 4 years older at diagnosis and more likely to be postmenopausal (Chung et al., 2013).

*Genetic risk factors*

Several of the studies investigated the association between gene polymorphisms and BC risk. In addition, there were also studies investigating the association between BC risk and DNA methylation (Kuchiba et al., 2014), length of CAG repeats (of androgen receptor alleles) (Chen et al., 2014a), mitochondrial DNA content (Jiang et al., 2014), mitochondrial D-loop insertions (Tipirisetti et al., 2014), and telomere length (Qu et al., 2013). The studies included in this review identified several associations between BC risk and genes that encode apoptotic regulators (e.g. BIM) (Lin et al., 2015), regulators of DNA stability or repair (e.g. BRCA1, ERCC1, TP53) (Chen et al., 2013a; Liu et al., 2013b; Pei et al., 2014; Sun et al., 2014a; Fu et al., 2015; Haryono et al., 2015; Wu et al., 2015), transcription factors (e.g. CDX2, TBX21) (Tulsyan et al., 2014; Yu et al., 2014; Iqbal et al., 2015), estrogen-related enzymes or receptors (e.g. CYP1A2, CYP19A1, ESR1) (Son et al., 2015; Sun et al., 2015b) and cell surface glycoproteins, growth factors or their receptors (e.g. IGKC, CD44, VEGF, IGF1R, HGF, FGFR2, ESR1) (Choi et al., 2014; Pandey et al., 2014; Siddiqui et al., 2014; Wang et al., 2014c; Yu et al., 2014; Son et al., 2015). Associations were also found for cytokines or regulators of cytokine signaling (e.g. SOCS4, IL6) (Choi et al., 2014; Joshi et al., 2014a), proteins involved in regulating circadian rhythm (e.g. PER3) (Wirth et al., 2014), a ligand-gated ion channel (e.g. CHRNA9) (Hsieh et al., 2014), or transcription factors involved in embryonic stem cell proliferation, renewal, and pluripotency (e.g. NANOG, OCT4) (Tulsyan et al., 2014). 

*Other risk factors for breast cancer in Asia*

Increased BC risk was associated with insulin use for 3 or more years (mortality risk) among participants from Thailand (Tseng, 2015); polycystic ovary syndrome (Shen et al., 2015); tonsillectomy (Sun et al., 2015a); sleep apnea (Chang et al., 2014); multiple sclerosis among participants from Taiwan (Sun et al., 2014c); inflammatory bowel disease among participants from Asia (Tsai et al., 2015); women who had an organ transplant (Pan et al., 2013); and diabetes among participants from China (Wang et al., 2013b). In addition, a study conducted in China found that hypertension was observed in a higher proportion of BC patients with type 2 diabetes compared with non-diabetic participants with BC (41.5 versus 26.1%, p= 0.001) (Wang et al., 2014b) demonstrating a link between breast cancer and other comorbidities. Another study conducted in China showed that premature ovarian failure was associated with decreased incidence of BC among postmenopausal women (Wu et al., 2014c). Higher levels of education have been linked to a reduction in breast cancer risk in Asia. (Lan et al., 2013)

Other investigated risk factors included hormone therapy (Lan et al., 2013), radiotherapy (Chitapanarux et al., 2013), BC-associated depression and anxiety (Wang et al., 2014a), environmental factors, including dichlorodiphenyltrichloroethane (DDT) exposure (Tang et al., 2014), and ratio of second to fourth finger length (Hong et al., 2014). 

**Table 1:** Summary of Incidence, Prevalence or Outcomes Data for Breast Cancer in Asia

Country	References	Reported data
China	Zhou HB, et al.(Zhou et al., 2015); Wu LZ, et al.(Wu et al., 2014b); Shi XJ, et al.(Shi et al., 2014b); Zhang X, et al.(Zhang et al., 2013b); Li XP, et al.(Li et al., 2013b)	- Crude incidence rate of 20.0 / 100,000 population between 2007 and 2012. - The age-standardized rate was 21.1 / 100,000 in 2012. - Average annual percentage change of 11.3%. - Mortality rate has increased yearly since 1991, a trend predicted to continue over the following 5 years. - After median 60 months of follow-up (range, 8–60 months), local and distant recurrence of breast cancer was observed in 23.4% of cases. - Between 2002 and 2006, the 5-year relative survival rate for breast cancer was > 40%. In Jiangsu between 2006 and 2010: - 11,013 new cases of female breast cancer and 3,068 deaths due to female breast cancer were identified. - The annual average crude incidence and age-standardized incidence were 25.2 and 17.9 per 100,000, and mortality rates were 7.03 and 4.81 per 100,000 respectively. - Incidence and mortality increased with annual percentage changes of 4.47% and 6.89%, respectively.
Japan	Hori M, et al.(Hori et al., 2015); Sugawara Y, et al.(Sugawara et al., 2013); Kasahara Y, et al.(Kasahara et al., 2013); Yamashiro H, et al.(Yamashiro et al., 2014); Matsuda A, et.al.(Matsuda et al., 2014); Wada K, et.al.(Wada et al., 2014)	- Leading type of cancer in females. - Primary invasive cancer incidence 775,601 in 2009. - Of 26,680 women, 148 incident cases of breast cancer were identified during 11 years of follow-up. - The rate of breast cancer detected in women aged 40–49 years was 0.28%. - Bone metastases developed in 11.3% (n=193) of recently diagnosed breast cancer cases - The bone-metastasis-free rate at 5 years was 89.2%.
Hong Kong	Kwong A, et al.(Kwong et al., 2014); Wong IO, et al.(Wong et al., 2015)	- Mean age at diagnosis of breast cancer in male and female patients was 64.5 and 52.7 years, respectively. - The 5-year OS, breast-cancer-specific mortality, and DFS for male and female patients were 78.7%, 90.5%, 90.5%, and 77.9%, 86.4%, and 81.4% respectively. - Age-standardized incidence projected to increase from 56.7 per 100,000 women in 2011–2015 to 62.5 per 100,000 women in 2021–2025. - Age-standardized mortality projected to decrease from 9.3 to 8.6 per 100,000 women for the same periods.
Korea	Park YH, et al.(Park et al., 2015b); Kim JH, et al.(Kim et al., 2014); Jung M, et al.(Jung et al., 2013)	- During a median follow-up of 100 months, the 5-year RFS rate and OS rate were 90.8% and 94.6%, respectively. - The 10-year estimated RFS rate and OS rate were 81.9% and 86.9%, respectively. - After median follow-up of 82 months, 5-year local RFS was 98.4% and the 10-year local RFS was 95.8% for all patients. Nineteen patients (2.6%) had ipsilateral breast recurrences, including 12 invasive recurrences and seven DCIS. Nine patients (1.2 %) patients developed contralateral breast cancer, including six invasive breast cancer and three DCIS. - The observed 10-year OS, breast cancer-specific survival and event-free survival was 61.4%, 62.3% and 59.1%, respectively.
Thailand	Virani S, et al.(Virani et al., 2014); Kotepui M, et.al.(Kotepui et al., 2014)	- Age-adjusted incidence rate increased by almost 300% from 1990 to 2010. - Incidence rates increased from 10.0 to 27.8 cases per 100,000 person-years. - Benign breast disease and fibroadenoma occurred in women aged < 40 years whereas breast cancer occurred in women aged > 40 years (OR: 8.629; 95% CI: 6.939–10.729; P < 0.001 and OR: 23.906; 95% CI: 18.359–31.129; P < 0.001, respectively). - Fibrocystic change occurred in patients aged < 40 years whereas breast cancer commonly occurred in patients aged ≥ 40 years (OR: 3.865; 95% CI: 2.993–4.991; P < 0.001).
India	Asthana S, et al.(Asthana et al., 2014)	- The annual percentage change in incidence for breast cancer ranged from 0.46% to 2.56%.
Asia	Keramatinia A, et al.(Keramatinia et al., 2014)	- 23,661 cases of breast cancer occurred in the 10 analyzed registries over 32 years of follow-up (1970–2002).
Malaysia	Hassan N, et al.(Hassan et al., 2015)	- Of the Chinese women in this study, 99.2% had a 10-year risk of breast cancer of < 2%.
Vietnam	Lan NH, et al.(Lan et al., 2013)	- OS rates at 1, 3, and 5 years following diagnosis were 0.94, 0.83 and 0.74 respectively.

DFS, disease-free survival; OS, overall survival; RFS, recurrence-free survival.

**Table 2:** Summary of Studies Reporting Risk Factors and Protective Factors for Breast Cancer in Asia

Factor		Studies		Comparison	Reported risk (odds ratio, relative risk or hazard ratio [95% Confidence interval])		Effect measure	
				RISK FACTORS				
Non-modifiable								
Sclerosing adenosis		Chen JJ, et al.(Chen et al., 2014b)		No comparator – retrospective file review of operable bilateral breast cancer cases.	11.8 (5.3, 26.3)		HR	
Inflammatory bowel disease requiring hospitalization more than twice per year		Tsai MS, et al.(Tsai et al., 2015)		No inflammatory bowel disease symptoms vs inflammatory bowel disease symptoms	8.45 (4.64, 15.4)		HR	
Ductal papilloma		Pan L, et al.(Pan et al., 2013)		Normal breast ultrasound vs abnormal breast	6.52 (1.87, 22.75)		OR	
Older age at menopause		Shamsi U, et al.(Shamsi et al., 2013)		Breast cancer vs no breast cancer	Age < 45 years 3.92 (2.52, 6.18) Age > 45 years 6.42 (3.47, 11.98)		OR	
Age > 40 years		Kotepui M, et al.(Kotepui and Chupeerach, 2013)		No comparator – retrospective file review of all breast cancer cases.	3.87 (2.99, 4.99)		OR	
Lobular carcinoma component involvement		Chen JJ, et al.(Chen et al., 2014b)		No comparator – retrospective file review of operable bilateral breast cancer cases.	5.6 (2.6, 12.1)		HR	
Diabetes		Chen YK, et al.(Chen et al., 2013b)		Graves’ disease vs no Graves’ disease	3.35 (1.02, 11.01)		OR	
Multiple sclerosis		Sun LM, et al.(Sun et al., 2014c)		Multiple sclerosis vs no Multiple sclerosis	2.23 (1.11, 4.46)		HR	
Family history of breast cancer		Zhou W, et al.(Zhou et al., 2013) Chen JJ, et al.(Chen et al., 2014b)		No comparator – retrospective file review of operable bilateral breast cancer cases.	2.11 (1.26, 3.52) First-degree relatives 2.0 (1.1, 3.4)		OR HR	
Polycystic ovary syndrome		Shen CC, et al.(Shen et al., 2015)		Polycystic ovary syndrome vs no Polycystic ovary syndrome	1.99 (1.05, 3.77)		HR	
Modifiable								
Demographics								
Low economic status		Kim SH, et al.(Kim et al., 2013)		No comparator – descriptive study	2.22 (not reported)		OR	
Single marital status		Shamsi U, et al.(Shamsi et al., 2013)		Breast cancer vs no breast cancer	1.55 (1.10, 2.39)		OR	
Married		Lan NH, et al.(Lan et al., 2013)		No comparator – retrospective data review	1.59 (1.09, 2.33)		HR	
Age < 20 years at first birth		Lee JS, et al.(Lee and Oh, 2014)		No comparator – retrospective data review	Luminal A 1.61 (0.62, 4.26)		HR	
Parity > 5		Lee JS, et al.(Lee and Oh, 2014)		No comparator – retrospective data review	Luminal A 1.95 (0.96, 3.97) Luminal B 1.12 (0.42, 3.02)		HR	
Not breastfeeding		Sugawara Y, et al.(Sugawara et al., 2013)		Mixed feeding vs formula feeding	Mixed feeding 1.12 (0.92, 1.37) Formula only 1.80 (1.14, 2.86)		HR	
Lifestyle								
Past or current smoker		Kim SH, et al.(Kim et al., 2013)		No comparator – descriptive study	3.77 (not reported)		OR	
Obesity		Noh HM, et al.(Noh et al., 2013)		Breast cancer vs no breast cancer	Postmenopausal 2.24 (1.22, 4.10)		OR	
Less than 1,000kcal of physical activity expenditure per week		Wang L, et al.(Wang et al., 2013a)		Breast cancer vs no breast cancer	2.17 (1.39, 3.39)		OR	
Nulliparity		Balasubramaniam SM, et al.(Balasubramaniam et al., 2013)		Breast cancer vs no breast cancer	2.4 (1.14, 5.08)		OR	
Insulin use ≥ 3 years		Tseng CH.(Tseng, 2015)		No comparator- data from National Health Insurance Program.	2.01 (1.1, 3.65)		HR	
Passive smoking		Pimhanam C, et al.(Pimhanam et al., 2014)		Breast cancer vs no breast cancer	3.77 (1.11. 12.82)		OR	
		Tong JH, et al.(Tong et al., 2014)		Breast cancer vs no breast cancer	1.46 (1.05, 2.03)		OR	
		Gao CM, et al.(Gao et al., 2013)		Breast cancer vs no breast cancer	1.47 (1.18, 1.84)		OR	
Factor		Studies		Comparison	Reported risk (odds ratio, relative risk or hazard ratio [95% Confidence interval])		Effect measure	
Increased BMI		Wang XL, et al.(Wang et al., 2013b) Sangrajrang S, et al.(Sangrajrang et al., 2013)		Breast cancer vs no breast cancer Breast cancer vs no breast cancer	1.58 (1.14, 2.19) >25mg/m2 1.33 (1.07, 1.65)		OR	
High perceived stress		Wang L, et al.(Wang et al., 2013a)		Breast cancer vs no breast cancer	1.65 (1.10, 2.47)		OR	
Dietary Intake									
Crucian carp	Gao CM, et al.(Gao et al., 2014)		Breast cancer vs no breast cancer			6.09 (3.04, 12.2)		OR	
Consumption of >30g fat/day	Balasubramaniam SM, et al.(Balasubramaniam et al., 2013)		Breast cancer vs no breast cancer			2.4 (1.14, 5.45)		OR	
Dietary PUFA	Kiyabu GY, et al.(Kiyabu et al., 2015)		Breast cancer vs no breast cancer			2.94 (1.26, 6.89)		HR	
Consumption of oils high in saturated fat	Balasubramaniam SM, et al.(Balasubramaniam et al., 2013) Sangrajrang S, et al.(Sangrajrang et al., 2013)		Breast cancer vs no breast cancer Breast cancer vs no breast cancer			2.0 (1.03, 4.52) Not reported		OR	
Dietary cadmium	Itoh H, et al.(Itoh et al., 2014) Nagata C, et al.(Nagata et al., 2013)		Breast cancer vs no breast cancer Breast cancer vs no breast cancer			1.94 (1.04, 3.63) Per 1.0ug/g increase in urinary cadmium level 1.67 (1.39, 2.01)		OR	
Sugar intake	Sulaiman S, et al.(Sulaiman et al., 2014)		Breast cancer vs no breast cancer			Premenopausal 1.93 (1.53, 2.61) Postmenopausal 1.87 (1.03, 2.61)		OR	
Meat Intake	Ko KP, et al.(Ko et al., 2013)		BRCA mutation vs no BRCA mutation			BRCA carriers 1.97 (1.13, 3.44)		HR	
High intake of fried and	Wang L, et al.(Wang et al., 2013a)		Breast cancer vs no breast cancer			1.86 (1.24, 2.77)		OR	
stir-fried food									
Vitamin D deficiency	Park S, et al.(Park et al., 2015a) Shi L, et al.(Shi et al., 2014a)		Breast cancer vs no breast cancer No comparator – retrospective study			1.27 (1.15, 1.39) Smoking 2.78 (1.11, 6.95) BMI <23 1 kg/m2 (reference) BMI 23-<2.5 kg/m2 1.12 (0.85, 1.47) BMI ≥27.5 kg/m2 1.57 (1.02, 2.25)		OR OR OR	
			PROTECTIVE FACTORS						
Imaging									
Normal breast ultrasound	Pan L, et al.(Pan et al., 2013)		Normal breast ultrasound vs abnormal breast ultrasound			0.14 (0.09, 0.22)		OR	
Lifestyle and demographic factors									
Higher Healthy Index score	Shahril MR, et al.(Shahril et al., 2013)		Breast cancer vs no breast cancer			Premenopausal 0.34 (0.15, 0.76) Postmenopausal 0.20 (0.06, 0.63)		OR	
Underweight BMI	Sangrajrang S, et al.(Sangrajrang et al., 2013)		Breast cancer vs no breast cancer			At 10 years old 0.70 (0.56, 0.88) At 20 years old 0.74 (0.59, 0.93)		OR	
Regular exercise	Sangrajrang S, et al.(Sangrajrang et al., 2013)		Breast cancer vs no breast cancer			0.78 (0.68, 0.98)		OR	
Literacy	Lan NH, et al.(Lan et al., 2013)		No comparator – retrospective data review			0.52 (0.89, 0.96)		HR	
Parity and Ovarian Health									
1-3 child births	Lee JS, et al.(Lee and Oh, 2014)		No comparator – retrospective data review			Luminal A recurrence 0.56 (0.34, 0.91) Luminal B recurrence 0.32 (0.17, 0.61)		HR	
Increasing parity	Shamsi U, et al.(Shamsi et al., 2013)		Breast cancer vs no breast cancer			0.90 (0.85, 0.97)		OR	
Premature ovarian failure	Wu X, et al.(Wu et al., 2014c)		Premature ovarian failure vs no premature ovarian failure			0.59 (0.38, 0.91)		OR	
Drug treatments or supplementation									
Hormone therapy	Lan NH, et al.(Lan et al., 2013)		No comparator – retrospective data review			0.22 (0.12, 0.41)		HR	
Vitamin D supplementation	Shamsi U, et al.(Shamsi et al., 2013)		Breast cancer vs no breast cancer			< 3 years 0.30 (0.12, 0.81) > 3 years 0.27 (0.13, 0.56)		OR	
Use of calcitonin nasal spray for osteoporosis	Sun LM, et al.(Sun et al., 2014d)		Osteoporosis and cancer vs osteoporosis and no cancer			0.35 (0.15, 0.80)		OR	
EPA (eicosapentaenoic acid)	Kiyabu GY, et al.(Kiyabu et al., 2015)		Breast cancer vs no breast cancer			0.47 (0.25, 0.89)		HR	
Factor	Studies		Comparison			Reported risk (odds ratio, relative risk or hazard ratio [95% Confidence interval])		Effect measure	
Dietary Intake									
Dietary fiber	Sulaiman et.al. (Sulaiman et al., 2014)		Breast cancer vs no breast cancer			Premenopausal 0.31 (0.12, 0.79) Postmenopausal 0.23 (0.07, 0.76)		OR	
Freshwater fish (black and silver carp)	Gao et.al. (Gao et al., 2014)		Breast cancer vs no breast cancer			Black carp 0.54 (0.33, 0.92) Silver carp 0.19 (0.11, 0.33)		OR	
Choline & betaine	Zhang et.al. (Zhang et al., 2013a)		Breast cancer vs no breast cancer			Choline 0.40 (0.28, 0.57) Betaine 0.58 (0.42, 0.80) Combined choline & betaine 0.38 (0.27, 0.53)		OR	
Soy products & isoflavone	Ko et. al.(Ko et al., 2013)		BRCA mutation vs no BRCA mutation			Soy in BRCA carriers 0.39 (0.19, 0.79)		HR	
	Li et. al. (Li et al., 2013a)		Breast cancer vs no breast cancer			Isoflavone Patients 0.53 (0.33, 0.85)		OR	
	Wada K, et al.(Wada et al., 2013)		Breast cancer vs no breast cancer			Isoflavone Population Controls 0.43 (0.26, 0.71)		OR	
Carotenoids	Wang et al. (2014a)		Breast cancer vs no breast cancer			a-carotene 0.61 (0.43, 0.88) b-carotene 0.54 (0.38, 0.78) b-cryptoxanthin 0.38 (0.26, 0.52) lutein / zeaxanthin 0.49 (0.34, 0.71)		OR	

## Discussion

*Non-modifiable risk factors*

The key risk factors for BC are non-modifiable, the strongest of which is being female (American Cancer Society, 2016). Similarly to the studies in Western population (American Cancer Society, 2016), advancing age has also been identified as a non-modifiable risk factor for developing BC in participants from Asia (Chen et al., 2014b; Kotepui et al., 2014; Virani et al., 2014). The studies identified two periods in which an increased BC risk emerged; first period at approximately 40–60 years, and a second peak at 85 years and over (Kotepui and Chupeerach, 2013; Chen et al., 2014b; Kotepui et al., 2014; Krishnatreya et al., 2014; Virani et al., 2014; Wu et al., 2014b; Varughese et al., 2015), which may be an important consideration for health service planning. 

Approximately 5–10% of BC cases are thought to arise from gene mutations; the most common cause of hereditary BC is an inherited mutation in the *BRCA1* and *BRCA2 *genes (American Cancer Society, 2016). Although the lifetime risk of BC among those with *BRCA1* mutations can be as high as 80%, individuals with mutations in *BRCA1* or *BRCA2* generally have a 55–65% or 45% increased risk of BC, respectively (National Cancer Institute, 2017). In 2016, the Asian BRCA consortium released its first paper on the management of hereditary breast and ovarian cancer (HBOC) in 14 Asian nations. This paper highlighted the disparity in the management of HBOC screening (only approximately 4,000 cases of BC in Asia had benefited from genetic services) between nations and recommended that policy makers, healthcare sectors and researchers address the limitations in HBOC testing and management (Nakamura et al., 2016). Results of the studies included in this review which considered the role of *BRCA* mutation in BC risk indicated that women with *BC* and a *BRCA1* mutation had an earlier age of disease onset, higher nuclear grade of BC, and a younger age of hospitalization (Chen et al., 2013a; Liu et al., 2013b; Pei et al., 2014; Sun et al., 2014a; Fu et al., 2015; Haryono et al., 2015; Wu et al., 2015) when compared to those without the *BRCA* mutation. Other gene mutations can also lead to* BC*, for example, *CHEK2*, *PTEN*, *CGH1*, *STK11* and* PALB2* (American Cancer Society, 2016). However, they are generally viewed as being much less common and most do not increase the risk of BC to the degree seen with the *BRCA* genes; other non-*BRCA* mutations have also been linked to increased BC. Interestingly, certain gene variations were associated with decreased BC risk (*e.g. RAD52, OCT4, FASL, IGFIR, APE1, BARD1, IL4, IL21*) (Kang et al., 2013; Liu et al., 2013a; You et al., 2013; Joshi et al., 2014b; Kang et al., 2014; Tulsyan et al., 2014; Xu et al., 2014; Wu et al., 2015), or both increased and decreased risk, depending on the gene variation investigated (e.g. *MTHFR, ESR1, VEGF, FAS, OGG1, TGFB1*) (Pooja et al., 2013; Kapahi et al., 2014; Lu et al., 2014; Luo et al., 2014; Wang et al., 2014c; Xu et al., 2014; Yu et al., 2014; Lu et al., 2015; Son et al., 2015).

Many women with BC do not have a family history of the disease. However, having a first-degree relative with BC is associated with a two-fold increase in risk of BC, and having two first-degree relatives can increase the risk three-fold (American Cancer Society, 2016). Accordingly, we found that family history of BC was associated with an increased risk in studies conducted among participants from China (Zhou et al., 2013; Chen et al., 2014b; Lee et al., 2014; Pei et al., 2014), Pakistan (Shamsi et al., 2013), Hong Kong (Tse et al., 2015), India (Bhadoria et al., 2013), and Korea (Park et al., 2013). 

Compared with other races or ethnic origins, Asian, Hispanic and Native American women may have a decreased risk of developing BC (American Cancer Society, 2016). This was supported by two studies which identified a reduced risk of BC among women from Asia and Malaysia compared with populations of women in the US (Horne et al., 2015; Sung et al., 2015). 

Early menarche (before age 12 years) and late menopause (after age 55 years) are associated with increased risk of BC, likely due to a longer lifetime exposure to estrogen and progesterone (American Cancer Society, 2016). Several studies demonstrated a link between menopausal status and BC risk and was seen across participants from Thailand (Anothaisintawee et al., 2014), India (Chattopadhyay et al., 2014), Pakistan (Song et al., 2014a), and Korea (Park et al., 2013). As expected, the studies showed an increased BC risk in women who had a lower age of menarche (Bhadoria et al., 2013; Chung et al., 2013) and a later age of menopause (Bhadoria et al., 2013; Shamsi et al., 2013; Lee et al., 2014). 

An increased risk of BC was identified in participants with pre-existing medical conditions including multiple sclerosis and polycystic ovary syndrome (Sun et al., 2014c; Shen et al., 2015).

*Modifiable risk factors*

Being overweight or obese is associated with an increased risk of BC, especially in women who have reached menopause. This is due to body fat, rather than the ovaries, becoming the primary source of estrogen in menopause (American Cancer Society, 2016). Internationally, several epidemiological studies have found that postmenopausal women with an increased BMI are at an increased BC risk of between 10–60% (Tahergorabi et al., 2016). In addition, being overweight or obese can increase insulin levels, and higher insulin levels have also been associated with increased risk of BC (American Cancer Society, 2016). Several of the studies in this review showed a correlation between increased BC risk and a high BMI, being overweight or obese, and low level of physical activity (Mizoo et al., 2013; Sangrajrang et al., 2013; Suzuki et al., 2013; Wang et al., 2013a; Wang et al., 2013b; Anothaisintawee et al., 2014; Wada et al., 2014; Fu et al., 2015). In addition one study showed that underweight BMI at a young age (at ages 10 and 20 years) were associated with a decreased risk of BC (Sangrajrang et al., 2013). 

In parallel with these observations, physical activity (walking) was associated with decreased risk of BC (Sangrajrang et al., 2013), although the exact amount of exercise needed to induce a protective effect remains unclear (American Cancer Society, 2016). 

The connection between BC, diabetes and obesity, was considered in a number of the identified studies. Breast cancer mortality risk was increased in women who had used insulin for more than 3 years (Tseng, 2015); additionally BC risk was associated with the presence of diabetes (Wang et al., 2013b). when compared to non-tamoxifen users (Sun et al., 2014b). Hypertension was observed in a higher proportion of BC patients with type 2 diabetes when compared to non-diabetic participants with BC (Wang et al., 2014b).

Women who have not had children or who had their first child after age 30 may have a slightly higher BC risk, whereas having many pregnancies and becoming pregnant at an early age may reduce the risk of BC (American Cancer Society, 2016). This was supported by some of the identified studies, in which BC risk increased when first full-term pregnancy was at an age greater than 25 years. Some studies also found that a later age at first live birth, first pregnancy and last pregnancy/birth also increased BC risk (Bhadoria et al., 2013; Huang et al., 2014; Pei et al., 2014). BC risk was found to decrease in women with history of full-term pregnancy (Pei et al., 2014). However, one study noted that increased parity and early age at first birth were associated with increased risk of BC recurrence (Lee and Oh, 2014).

Diet and the use of vitamins are modifiable risk factors with an unclear effect on BC. High consumption of fruits and vegetables was associated with decreased BC risk (Sangrajrang et al., 2013), and high intake of fatty foods was associated with increased BC risk (Balasubramaniam et al., 2013; Wang et al., 2013a). Data suggested that an intake of vitamin D, dietary fiber and intake of plant- and soy-based products may be protective for BC (Ko et al., 2013; Li et al., 2013a; Wada et al., 2013; Shi et al., 2014a; Sulaiman et al., 2014; Wang et al., 2014a; Park et al., 2015a), whereas high intake of dietary salt, sugar and meat may increase the risk of BC (Ko et al., 2013; Park et al., 2014b; Sulaiman et al., 2014).

There is increasing evidence which supports the link between BC risk and heavy smoking over a long period of time (American Cancer Society, 2016). This was confirmed in several articles demonstrating a clear association between BC risk and exposure, either active or passive, to tobacco smoke (Mizoo et al., 2013; Pimhanam et al., 2014; Tong et al., 2014; Wada et al., 2015). 

## Limitations

There are several limitations to this study. Firstly, due to the large number of articles identified, the results included are based only on the content of article abstracts, as assessed by a single reviewer. Therefore, the results analysis is limited due to some missing data on comparators, effect sizes and other statistical analyses. The missing data also make it difficult to prioritize results according to the strength or quality of included studies and/or data. The strength of the article may also be limited due to the absence of methods to assess risk of bias within individual studies and across studies, and the absence of additional analyses of the extracted data.

In conclusions, this literature review has underscored the importance and relevance of known non-modifiable and modifiable risk factors for Asian women with BC. Furthermore, it has highlighted the differences in epidemiology of BC in Asian nations when compared to other regions. Only a few studies directly compared BC risk between Asian and non-Asian populations; in the ones that did, there was a decreased risk of BC among women from Asia compared with women from other countries such as the US. 
